# Supplementary material for: Dynamical Modeling of Behaviorally Relevant Spatiotemporal Patterns in Neural Imaging Data
Source: ArXiv. 2025 Sep 23:arXiv:2509.18507v1. Preprint. [Version 1] (PMC12486059)
Supplement: Supplement 1 [file NIHPP2509.18507v1-supplement-1.pdf]

A. Appendix

A.1. Methods Supplementary Details

A.1.1. Formulation of the Full Inference Model

Our model employs a two-RNN architecture to effectively capture and disentangle behaviorally relevant neural dynamics from other neural dynamics. The first RNN (*ConvRNN1*) focuses on behaviorally relevant dynamics before the second RNN (*ConvRNN2*) learns the remaining dynamics.

ConvRNN1 is parameterized by $\boldsymbol{f}_{\boldsymbol{A}}^{(1)}(\cdot)$, $\boldsymbol{K}^{(1)}(\cdot)$, $\boldsymbol{C}^{(1)}(\cdot)$, and $\boldsymbol{D}^{(1)}(\cdot)$. It takes as input the neural images, $\mathbf{Y}_k$, and outputs a latent state representation, $\mathbf{X}_k^{(1)} \in \mathbb{R}^{n_1 \times H' \times W'}$, that captures the behaviorally relevant neural dynamics.

ConvRNN2 is parameterized by $\boldsymbol{f}_{\boldsymbol{A}}^{(2)}(\cdot)$, $\boldsymbol{K}^{(2)}(\cdot)$, and $\boldsymbol{C}^{(2)}(\cdot)$. It takes as input the neural images, $\mathbf{Y}_k$, as well as the latent states from *ConvRNN1*, $\mathbf{X}_k^{(1)}$, and outputs a latent state representation, $\mathbf{X}_k^{(2)} \in \mathbb{R}^{n_2 \times H' \times W'}$, that captures the residual neural dynamics not captured by *ConvRNN1*.

In Equation 2, both latent states were combined for simplicity. The full inference model (Figure 1a) can be formulated as follows:

$$\begin{cases} \mathbf{X}_{k+1}^{(1)} &= \boldsymbol{f}_{\boldsymbol{A}}^{(1)}(\mathbf{X}_k^{(1)}) + \boldsymbol{K}^{(1)}(\mathbf{Y}_k) \\ \mathbf{X}_{k+1}^{(2)} &= \boldsymbol{f}_{\boldsymbol{A}}^{(2)}(\mathbf{X}_k^{(2)}) + \boldsymbol{K}^{(2)}(\mathbf{Y}_k, \mathbf{X}_{k+1}^{(1)}) \\ \hat{\mathbf{Y}}_k &= \boldsymbol{C}^{(1)}(\mathbf{X}_k^{(1)}) + \boldsymbol{C}^{(2)}(\mathbf{X}_k^{(2)}) \\ \hat{\mathbf{z}}_k &= \boldsymbol{D}^{(1)}(\mathbf{X}_k^{(1)}) \end{cases} \tag{A.1}$$

where $\hat{\mathbf{Y}}_k \in \mathbb{R}^{n_y \times H \times W}$ is the predicted neural images and $\hat{\mathbf{z}}_k \in \mathbb{R}^{n_z}$ is the predicted behavior at time index, k.

The full set of states can be denoted as $\mathbf{X}_k \in \mathbb{R}^{n_x \times H' \times W'}$ where $n_x = n_1 + n_2$, and is achieved by concatenating the image latent states in the channel dimensions. As seen in Equation A.1, $\mathbf{X}_k^{(1)}$ is calculated independently of $\mathbf{X}_k^{(2)}$. As discussed in Section A.1.4, $\mathbf{X}_k^{(1)}$ are learned to decode behavior, so they essentially capture behaviorally relevant neural dynamics. $\mathbf{X}_k^{(2)}$ learn other neural dynamics by optimizing for neural prediction. This is achieved by passing the neural images and states from *ConvRNN1* as residuals in the calculation of the second set of states. The above formulation can be written in combined form as in Equation 2, where:

$$\begin{aligned} \mathbf{X}_k &= \begin{bmatrix} \mathbf{X}_k^{(1)} & \mathbf{X}_k^{(2)} \end{bmatrix}^T, \\ \boldsymbol{f}_{\boldsymbol{A}}(\mathbf{X}_k) &= \begin{bmatrix} \boldsymbol{f}_{\boldsymbol{A}}^{(1)}(\mathbf{X}_k^{(1)}) \\ \boldsymbol{f}_{\boldsymbol{A}}^{(2)}(\mathbf{X}_k^{(2)}) \end{bmatrix} = \begin{bmatrix} \mathrm{GlobalAttn}^{(1)}(\boldsymbol{A}^{(1)} * \mathbf{X}_k^{(1)}) \\ \mathrm{GlobalAttn}^{(2)}(\boldsymbol{A}^{(2)} * \mathbf{X}_k^{(2)}) \end{bmatrix}, \\ \boldsymbol{K}(\mathbf{Y}_k) &= \begin{bmatrix} \boldsymbol{K}^{(1)}(\mathbf{Y}_k) \\ \boldsymbol{K}^{(2)}(\mathbf{Y}_k, \mathbf{X}_{k+1}^{(1)}) \end{bmatrix}, \\ \boldsymbol{C}(\mathbf{X}_k) &= \boldsymbol{C}^{(1)}(\mathbf{X}_k^{(1)}) + \boldsymbol{C}^{(2)}(\mathbf{X}_k^{(2)}), \\ \boldsymbol{D}(\mathbf{X}_k) &= \boldsymbol{D}^{(1)}(\mathbf{X}_k^{(1)}) \end{aligned}$$

These notations connect the full inference model (Equation A.1) and the combined form in Equation 2. Also, $\boldsymbol{f}_{\boldsymbol{A}}(.) = \mathrm{GlobalAttn}(\boldsymbol{A} * (.))$, where $\mathrm{GlobalAttn}$ represents the self-attention mechanism, and $\boldsymbol{A}$ represents the convolutional kernels applied on the states prior to self-attention.

A.1.2. Details of Self-Attention Operation

The recurrence function, $\boldsymbol{f}_{\boldsymbol{A}}^{(1)}(\cdot)$, is designed to capture spatiotemporal dependencies in the latent state representation of *ConvRNN1* both locally and globally (Figure A.1). For simplicity, we explain details of applying the recurrence function $\boldsymbol{f}_{\boldsymbol{A}}^{(1)}(\cdot)$, on the latent state at time index k, $\mathbf{X}_k^{(1)}$. However, the same function is applied at all other time indices recurrently.

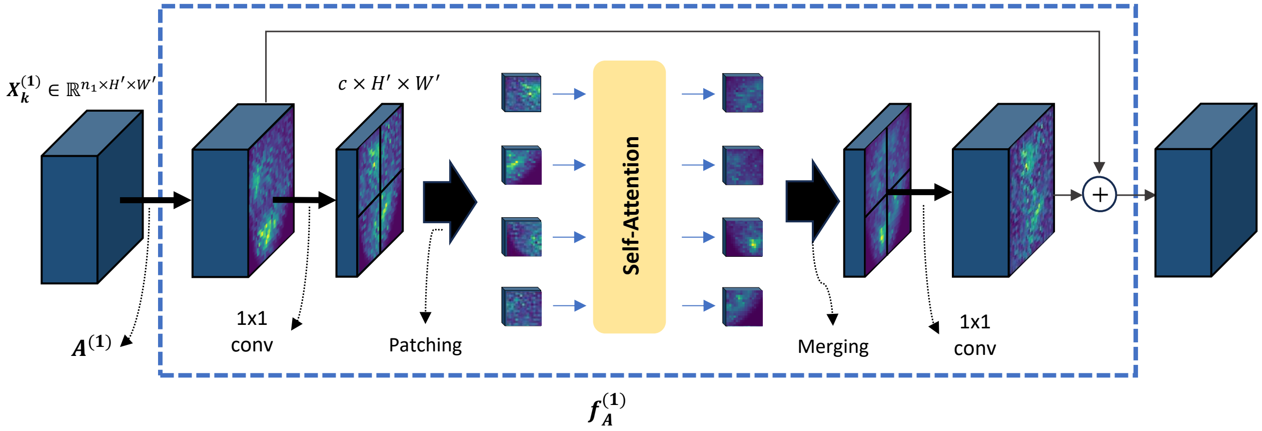

Figure A.1. Details of self-attention mechanism in $\boldsymbol{f}_{\boldsymbol{A}}^{(1)}$. Self-attention is used here to capture global spatial relationships within the latent state. $\boldsymbol{f}_{\boldsymbol{A}}^{(2)}$ applies the same self-attention mechanism to $\mathbf{X}_k^{(2)}$ using a different set of parameters

$\boldsymbol{A}^{(1)}$ is a single convolutional layer to capture local dependencies in the latent state. Additionally, the recurrence functions incorporate a self-attention mechanism to capture global context and long-range dependencies in the latent state dynamics. Here we explain the details of applying self-attention to $\mathbf{X}_k^{(1)}$.

Reduction in channel dimension. For $\boldsymbol{f}_{\boldsymbol{A}}^{(1)}(\cdot)$, the process begins by passing $\mathbf{X}_k^{(1)}$ through a 1x1 convolutional layer to reduce the channel size from n_x to c, resulting in a representation with dimensions $c \times H' \times W'$. This reduction in channel size is optional and makes subsequent self-attention computation more efficient. In our experiments, we found that reducing the channel dimension to $c = 2$ provided a good balance between computational and parameter efficiency, and model performance.

Patching. Next, the latent state representation is divided into patches, $\{\mathbf{x}_{k,1}^{(1)}, \mathbf{x}_{k,2}^{(1)}, ..., \mathbf{x}_{k,M}^{(1)}\}$, similar to the approach used in (Dosovitskiy et al., 2021). The total number of patches, M, is calculated as $M = (H' \times W')/(P \times P)$, where P is the patch size. Each patch, $\mathbf{x}_{k,i}^{(1)}$, has dimensions $c \times P \times P$ and can be interpreted as a representation of features from roughly a specific brain region.

Self-attention block. The patches are then flattened into vectors of shape cP^2 and treated as tokens for the self-attention mechanism. A one-dimensional learnable embedding is added to each of the tokens (patches), so the self-attention layer is informed of the position of the embedding in the latent state images. Multi-head self-attention is applied to these tokens (Vaswani et al., 2017), allowing the model to learn spatial relationships between different patches. layer normalization is applied before and after the self-attention.

Projecting back to the latent state representation. After applying self-attention to the embedded patches, the patches are reshaped and rearranged into $c \times H' \times W'$ to get to the original spatial dimensions, effectively reversing the patching operation. A 1×1 convolutional layer then projects the $c \times H' \times W'$ latents to an $n_1 \times H' \times W'$ dimensional space. The resulting representation is added to the output of $\boldsymbol{A}^{(1)}$, forming the final output of the recurrence function.

This combination of convolutional layers and self-attention layers in the recurrence function enables the model to effectively capture both local and global spatial dependencies. The same recurrence function is applied to $\mathbf{X}_k^{(2)}$, which has dimensions $n_2 \times H' \times W'$, across all time points using a different set of parameters to learn a separate set of dynamics for the behaviorally irrelevant component of the neural images.

A.1.3. Loss Functions

Neural Prediction Loss For neural prediction, we use a combination of L1, L2, and gradient difference loss (GDL) (Mathieu et al., 2016). The GDL loss encourages the preservation of local image structure by penalizing differences between

the gradients of the predicted and ground-truth images. This combined loss function aims to improve the accuracy and structural fidelity of the predicted neural images. The L1, L2, and GDL functions are defined as follows:

$$\mathcal{L}_{L1}(\hat{Y}_k, Y_k) = \sum_{i,j} |\hat{Y}_k^{i,j} - Y_k^{i,j}|, \tag{A.2}$$

$$\mathcal{L}_{L2}(\hat{Y}_k, Y_k) = \sum_{i,j} (\hat{Y}_k^{i,j} - Y_k^{i,j})^2, \tag{A.3}$$

$$\mathcal{L}_{grad}(\hat{Y}_k, Y_k) = \sum_{i,j} \big| ||Y_k^{i,j} - Y_k^{i-1,j}|| - ||\hat{Y}_k^{i,j} - \hat{Y}_k^{i-1,j}|| \big| + \big| ||Y_k^{i,j-1} - Y_k^{i,j}|| - ||\hat{Y}_k^{i,j-1} - \hat{Y}_k^{i,j}|| \big|, \tag{A.4}$$

where i, j index the spatial dimensions of the image. The total loss for neural image reconstruction is given by:

$$\mathcal{L}_{\mathbf{Y}} = \mathcal{L}_{L2}(\hat{Y}_k, Y_k) + \lambda_{L1}\mathcal{L}_{L1}(\hat{Y}_k, Y_k) + \lambda_{grad}\mathcal{L}_{grad}(\hat{Y}_k, Y_k), \tag{A.5}$$

where λ_{L1} and λ_{grad} are hyperparameters that control the relative weights of the L1 and GDL losses.

Behavior Decoding Loss For behavior decoding, the loss function, $\mathcal{L}_{\mathbf{z}}$, is chosen based on the distribution and availability of data at each time point.

- **Continuous Behavior:** Assuming an isotropic Gaussian distribution for this type of behavior, we use the MSE loss.
- **Categorical Behavior:** We use class-weighted cross-entropy loss for categorically distributed behavior to address potential class imbalance (Lin et al., 2017). For instance, in the WFCI 2 dataset, where the mouse is not touching the sensors over 90% of the time, we assign a weight of 0.9 to class 1 (indicating when the mouse is touching a sensor—left handle, right handle, left spout, or right spout) and 0.1 to class 0 (indicating when the mouse is not touching a sensor).
- **Intermittently Recorded Behavior:** We utilize a masking strategy to handle intermittently recorded behavior. The behavior loss is calculated only at the sparse time points where the behavior is observed.

A.1.4. Two-Phase Learning Details

To disentangle behaviorally relevant neural dynamics from other neural dynamics, we design a model architecture with two ConvRNNs, each incorporating self-attention mechanisms. The parameters of these two ConvRNNs are learned in two sequential phases to achieve the disentanglement.

Phase 1: Learning Behaviorally Relevant Dynamics

First, the parameters of *ConvRNN1* - i.e., $\boldsymbol{f}_{\boldsymbol{A}}^{(1)}(\cdot)$, $\boldsymbol{K}^{(1)}(\cdot)$, and $\boldsymbol{D}^{(1)}(\cdot)$ - and the behaviorally relevant latent states, $\mathbf{X}_k^{(1)}$, are learned to minimize the error in predicting behavior from the neural images. The following recurrent formula is used to predict behavior, $\hat{\mathbf{z}}_k^{(1)}$, one-step into the future:

$$\begin{cases} \mathbf{X}_{k+1}^{(1)} &= \boldsymbol{f}_{\boldsymbol{A}}^{(1)}(\mathbf{X}_k^{(1)}) + \boldsymbol{K}^{(1)}(\mathbf{Y}_k) \\ \hat{\mathbf{z}}_k^{(1)} &= \boldsymbol{D}^{(1)}(\mathbf{X}_k^{(1)}) \end{cases} \tag{A.6}$$

The optimization is formulated as:

$$\min_{\boldsymbol{f}_{\boldsymbol{A}}^{(1)}, \boldsymbol{K}^{(1)}, \boldsymbol{D}^{(1)}} \sum_k \mathcal{L}_{\mathbf{z}}(\mathbf{z}_k, \hat{\mathbf{z}}_k^{(1)}), \tag{A.7}$$

where $\mathcal{L}_{\mathbf{z}}$ is the loss function for behavior decoding chosen based on the distribution of behavior (Appendix A.1.3). $k \in [1, 2, ..., T]$ where T is the total number of samples. This optimization ensures that *ConvRNN1* learns neural dynamics that are relevant to behavior.

Once *ConvRNN1* is learned, its parameters are fixed, and the decoder $\boldsymbol{C}^{(1)}(\cdot)$ is optimized to predict neural images one step into the future from the learned latent states, $\mathbf{X}_k^{(1)}$. This can be formulated as:

$$\hat{\mathbf{Y}}_k^{(1)} = \boldsymbol{C}^{(1)}(\mathbf{X}_k^{(1)}) \tag{A.8}$$

and the optimization is formulated as:

$$\min_{\boldsymbol{C}^{(1)}} \sum_k \mathcal{L}_{\mathbf{Y}}(\mathbf{Y}_k, \hat{\mathbf{Y}}_k^{(1)}), \tag{A.9}$$

where $\mathcal{L}_{\mathbf{Y}}$ is the loss function for neural reconstruction, defined in Equation A.5.

Phase 2: Learning Residual Neural Dynamics

In this phase, the parameters of *ConvRNN2* - i.e., $\boldsymbol{f}_{\boldsymbol{A}}^{(2)}(\cdot)$, $\boldsymbol{K}^{(2)}(\cdot)$, and $\boldsymbol{C}^{(2)}(\cdot)$ - are learned to minimize the error in predicting the residual neural images, i.e., the part of neural images not predicted by the behaviorally relevant states of *ConvRNN1*. This is achieved by training *ConvRNN2* to predict the difference between the observed neural images and the neural images predicted by *ConvRNN1*. The residual neural predictions are calculated using the following recurrent formulation:

$$\begin{cases} \mathbf{X}_{k+1}^{(2)} &= \boldsymbol{f}_{\boldsymbol{A}}^{(2)}(\mathbf{X}_k^{(2)}) + \boldsymbol{K}^{(2)}(\mathbf{Y}_k, \mathbf{X}_{k+1}^{(1)}) \\ \hat{\mathbf{Y}}_k^{(2)} &= \boldsymbol{C}^{(2)}(\mathbf{X}_k^{(2)}) \end{cases} \tag{A.10}$$

This step learns the residual neural dynamics and the latent states, $\mathbf{X}_k^{(2)}$. The optimization is formulated as:

$$\min_{\boldsymbol{f}_{\boldsymbol{A}}^{(2)}, \boldsymbol{K}^{(2)}, \boldsymbol{C}^{(2)}} \sum_k \mathcal{L}_{\mathbf{Y}}(\mathbf{Y}_k - \hat{\mathbf{Y}}_k^{(1)}, \hat{\mathbf{Y}}_k^{(2)}) \tag{A.11}$$

This concludes learning the ConvRNNs and the total latent states $\mathbf{X}_k$. Note that in the two optimization steps in Equations A.9, and A.11, the optimization only controls and learns the parameters in the current optimization, and the parameters from previous optimizations are fixed.

A.1.5. Alternative Formulation for the Inference Model

We can optionally concatenate the mappings from the encoder, $\boldsymbol{K}(\mathbf{Y}_k)$, with the states of the current ConvRNN before feeding the latent states into the recurrence function. This can be formulated as:

$$\begin{cases} \mathbf{X}_{k+1}^{(1)} &= \boldsymbol{f}_{\boldsymbol{A}}^{(1)}(\mathbf{X}_k^{(1)}, \boldsymbol{K}^{(1)}(\mathbf{Y}_k)) \\ \mathbf{X}_{k+1}^{(2)} &= \boldsymbol{f}_{\boldsymbol{A}}^{(2)}(\mathbf{X}_k^{(2)}, \mathbf{X}_{k+1}^{(1)}, \boldsymbol{K}^{(2)}(\mathbf{Y}_k)) \\ \hat{\mathbf{Y}}_k &= \boldsymbol{C}^{(1)}(\mathbf{X}_k^{(1)}) + \boldsymbol{C}^{(2)}(\mathbf{X}_k^{(2)}) \\ \hat{\mathbf{z}}_k &= \boldsymbol{D}^{(1)}(\mathbf{X}_k^{(1)}) \end{cases} \tag{A.12}$$

This can be thought of as including the information from the neural images at the current time index within the mapping. This does not change the dimensions of the latent states, but the input to the convolutional layer within the recurrence uses more kernels. This is a more general form of Equation A.1, and for the first two datasets, this form achieves slightly better performance (See Table A.6).

A.1.6. SBIND Architecture and Implementation Details

The Neural Encoders, $\boldsymbol{K}^{(1)}$ and $\boldsymbol{K}^{(2)}$, each consist of three convolutional layers that downsample the input neural images to a 32×32 spatial resolution. These layers process the images statically and locally. To ensure stable learning, each convolutional layer is followed by batch normalization to normalize activations, and Leaky ReLU (Xu et al., 2015) is used as the activation function. Padding is applied to preserve spatial dimensions during convolutions.

The neural decoders, $\boldsymbol{C}^{(1)}$ and $\boldsymbol{C}^{(2)}$, consist of three transposed convolutional layers, which are designed to upsample the latent states and project them back into the neural image observation space. The same activation function and normalization are applied to these decoders.

The behavior decoder, $\boldsymbol{D}$, begins by further downsampling the 32×32 latent state to a 4×4 spatial resolution using three convolutional layers, each with stride 2, batch normalization, Leaky ReLU activation, and channel dropouts (Tompson et al., 2015). This reduction is followed by a fully connected layer to project the latent state into the behavior observation space.

The recurrence functions, $\boldsymbol{f}_{\boldsymbol{A}}^{(1)}$ and $\boldsymbol{f}_{\boldsymbol{A}}^{(2)}$, each use a single convolutional layer with 3×3 kernels to process the latent states locally. Afterward, the model applies multi-head self-attention to patches of the latent state images to capture long-range dependencies across the latent space. Each latent patch is mapped to a 256-dimensional embedding space, which is then used to compute the self-attention.

Training details, including the learning rate, optimizer choice, and hyperparameters of the mappings, are summarized in Table A.1.

Hyperparameter Tuning:

We use latent states with dimensions $n_x \times 32 \times 32$ across all experiments. The first n_1 channels of the latent states are used in *ConvRNN1* to model behaviorally relevant dynamics. Any additional channels are used for *ConvRNN2* to learn residual neural dynamics ($n_2 = \max(n_x - n_1, 0)$). We use $n_1 = 8$ across all experiments. The number of latent channels, n_x, was tuned in the set $\{1, 2, 4, 8, 16, 32\}$, where for $n_x \leq 8$ only *ConvRNN1* is trained, and for $n_x > 8$ *ConvRNN2* is learned in the second phase in addition to *ConvRNN1*. The patch size for the self-attention layer in $\boldsymbol{f}_{\boldsymbol{A}}^{(1)}$ and $\boldsymbol{f}_{\boldsymbol{A}}^{(2)}$ was tuned in the set $\{1, 2, 4, 8, 16\}$.

Implementation Details : For WFCI 1 dataset with 39200 neural image samples used for training, it takes 2445 seconds on average to run all 3 optimization steps for 80 epochs on an NVIDIA RTX 6000 Ada Generation GPU, and the inference takes 13.5 seconds on 9800 sequential samples.

A.2. Ablation Details

A.2.1. MLP (PARAMETERIZED)-SBIND

This ablation explores the importance of convolutional layers in our model by replacing them with MLPs. It maintains the same two-phase learning scheme as SBIND, but uses MLPs to parameterize all the mappings in both phases and relies on MLPs to learn spatiotemporal structure in neural image data. The states $\mathbf{X}_k^{(1)}$ and $\mathbf{X}_k^{(2)}$ are vectors of shape $\mathbb{R}^{n_1 \times 1 \times 1}$ and $\mathbb{R}^{n_2 \times 1 \times 1}$, respectively, disregarding the spatial distribution in neural images.

Optionally, we use commonly used preprocessing techniques on widefield and ultrasound data to obtain a low-dimensional representation (as a vector) for the neural images. This representation is then used as input for training and inference on the model. After learning the model and obtaining predictions in the low-dimensional space, we project the prediction back to the neural image space to compare the performance in neural prediction with SBIND.

A.2.2. SBIND W/O $\boldsymbol{f}_{\boldsymbol{A}}$

This ablation investigates the importance of recurrent neural networks in our model by removing the recurrence mapping, $\boldsymbol{f}_{\boldsymbol{A}}(\cdot)$. This is essentially a Convolutional Autoencoder (CAE) that takes neural images as input and attempts to predict neural images one-step into the future. This is equivalent to our model without the recurrence function, just optimizing for $\boldsymbol{K}$, $\boldsymbol{C}$, and $\boldsymbol{D}$. This ablation is used to pinpoint the importance of using information from neural images more than one sample in the past for modeling. A behavior decoder mapping, $\boldsymbol{D}$ is trained downstream to project the latent representation of the CAE, $\mathbf{X}_k$, to the behavior of interest. The same loss functions from SBIND are used for neural and behavioral prediction. The inference of this model can be formulated as:

$$\begin{cases} \mathbf{X}_{k+1} &= \boldsymbol{K}(\mathbf{Y}_k) \\ \hat{\mathbf{Y}}_{k+1} &= \boldsymbol{C}(\mathbf{X}_{k+1}) \\ \hat{\mathbf{z}}_{k+1} &= \boldsymbol{D}(\mathbf{X}_{k+1}) \end{cases} \tag{A.13}$$

Table A.1. SBIND Model and Training Details and Hyperparameters Across All Datasets

Component	Hyperparameter	WFCI 1	WFCI 2	fUSI
General	Input Dimensions	1x128x128	1x128x128	1x128x128
	n_1	8	8	8
	n_2	8	24	24
	Batch Size	7	7	30
	Sequence Length	63	63	30
	Learning Rate	1e-3	1e-3	1e-3
	LR Schedule	StepLR	StepLR	StepLR
	Max Training Epochs	80	80	80
	Weight Decay	1e-6	1e-6	1e-6
Neural Encoders $\boldsymbol{K}^{(1)}$ ($\boldsymbol{K}^{(2)}$)	Layers	3	3	3
	Kernel Size	5x5	5x5	5x5
	Strides	2, 2, 1	2, 2, 1	2, 2, 1
	Channel Dropout	0	0	0
	Num Kernels	32, 32, n_1 (n_2)	32, 32, n_1 (n_2)	32, 32, n_1 (n_2)
Neural Decoder $\boldsymbol{C}^{(1)}(\boldsymbol{C}^{(2)})$	Layers	2	2	2
	Kernel Size	5x5	5x5	5x5
	Strides	2	2	2
	Channel Dropout	0	0	0
	Num Kernels	32, 32, 1	32, 32, 1	32, 32, 1
	λ_{L1}	2.0	2.0	2.0
	λ_{grad}	0.3	0.3	0.3
Behavior Decoder ($\boldsymbol{D}^{(1)}$)	Conv Layers	3	3	4
	Kernel Size	5x5	5x5	5x5
	Strides	2	2	2
	Channel Dropout	0.4	0.4	0.4
	Num Kernels	64, 64, 64	64, 64, 64	16, 16, 16, 16
	FCN Layers	1	1	1
	FCN hidden units	64	64	16
Recurrence function $\boldsymbol{f}_{\boldsymbol{A}}^{(1)}$ ($\boldsymbol{f}_{\boldsymbol{A}}^{(2)}$)	Conv. Layers	1	1	1
	Kernel Size	3x3	3x3	3x3
	Strides	1	1	1
	Channel Dropout	0	0	0
	Hidden Dim	48	48	48
	Num Kernels	n_1 (n_2)	n_1 (n_2)	n_1 (n_2)
	Self-Attention Heads	8	8	8
	Patch Size	8	8	8
	Embedding Dim	256	256	256
	Positional Embedding	Learnable	Learnable	Learnable
	Num Patches	16	16	16

A.2.3. SBIND MSE $L_{\mathbf{Y}}$

This ablation explores the effect of the neural loss function in Equation A.5 by removing the GDL and L1 loss components. This ablation uses the same model architecture and training procedure as SBIND, but trains the model using only the MSE loss for neural prediction in the optimizations of Equations A.9 and A.11.

A.2.4. SBIND-UNSUP (UNSUPERVISED)

This ablation investigates the importance of disentangling behaviorally relevant dynamics by removing the first phase of our algorithm. This forces the model to learn all neural dynamics without considering their relevance to behavior, as it predicts neural data one step into the future without using behavior information. Effectively, this ablation sets $n_1 = 0$ and

$n_x = n_2$, as it learns $\mathbf{X}_k^{(2)}$ and *ConvRNN2* parameters while still using self-attention in the recurrence and convolutional layers. Because SBIND-Unsup only learns a single ConvRNN for neural prediction, it may learn behaviorally irrelevant neural dynamics in neural images, potentially resulting in inferior behavioral prediction performance.

We use the same latent state dimensions, $n_x \times H' \times W'$, whenever we compare the performance of SBIND with this variant.

A.2.5. SBIND NoAtt

This ablation assesses the impact of the self-attention mechanism by removing it from the recurrence function, $\boldsymbol{f_A}(\cdot)$. This model still disentangles behaviorally relevant neural dynamics using the two-phase learning scheme. It also uses the same hyperparameters for all other mappings ($\boldsymbol{K}$, $\boldsymbol{C}$, and $\boldsymbol{D}$) and the same loss functions as SBIND. However, it does not utilize self-attention to capture long-range spatial information in the latent space, and consequently, in the neural image space. By removing self-attention, $\boldsymbol{f_A}(\cdot)$ simplifies to a local convolutional layer. This prevents the model from capturing dependencies between distant brain regions and using these dependencies for neural and behavioral prediction (Figure 4).

A.3. Baseline Neural-behavioral Models and Preprocessing Methods

First, we list the two dimensionality reduction methods that are commonly used when working with widefield calcium and functional ultrasound imaging data. We use these as optional preprocessing steps to obtain a low-dimensional representation for neural-behavioral baselines. We compare our model with CEBRA (Schneider et al., 2023), which extracts latent embeddings informed by behavior and fits decoders for neural and behavior observations. Moreover, we compare our model performance with DPAD (Sani et al., 2024), which is a nonlinear dynamical model that learns behaviorally relevant neural dynamics.

A.3.1. PCA

PCA is often performed on widefield calcium imaging data as a dimensionality reduction technique before performing modeling (Musall et al., 2019). Enough principal components are extracted to explain a sufficient amount of variance in the neural images. For functional ultrasound imaging, PCA serves the same purpose and is also employed to decode movement intentions (Griggs et al., 2024; Norman et al., 2021).

When using PCA for preprocessing the baseline models, we tune the number of principal components (PCs) used to represent the neural images, treating it as a hyperparameter. We select the number of PCs from the set of values $\{25, 50, 100, 200, 400\}$. After training baseline models with PCA preprocessing and obtaining predictions of PCs in the low-dimensional space, we project the predictions back to the neural image space to compare the performance in neural prediction with SBIND.

A.3.2. LocaNMF

Localized semi-nonnegative matrix factorization (LocaNMF) (Saxena et al., 2020) is a dimensionality reduction method that decomposes widefield imaging data into localized spatial components and corresponding temporal components. The temporal component is used as a low-dimensional representation of widefield calcium imaging data. LocaNMF leverages the Allen brain atlas (Wang et al., 2020) to initialize spatial components and encourages localization by limiting their spread, while still allowing contributions from neighboring regions to capture relevant variance. The result is a more interpretable decomposition, where each temporal component primarily corresponds to a specific brain region

When using LocaNMF for preprocessing, we tune its hyperparameters. The number of components for Singular Value Decomposition is varied within the set of values $\{100, 200, 400, 1000\}$. The "minrank" hyperparameter is selected from the set $\{1, 2, 5\}$. Other hyperparameters are set to their default values.

A.3.3. CEBRA

CEBRA (Schneider et al., 2023) is a non-dynamic model that uses convolutional neural network encoders in its architecture to process neighboring time points of the data within a small, fixed window length. It uses a contrastive loss to extract latent embeddings informed by either simultaneous behavior labels or time information. CEBRA-Behavior uses an objective that aligns neural activity in the embedding space such that time points with similar behavior have similar embeddings. CEBRA-Time uses time information to extract the embeddings. After learning the embeddings, it fits decoders to map the embeddings from each time point to the observation space (i.e., behavior or neural).

We performed hyperparameter tuning for CEBRA. We used the default "KNN-Decoder" for categorical behavior prediction in WFCI 2 and fUSI datasets. For neural prediction and continuous behavior, we tested the default "KNN-Decoder", "L1 Linear Regressor", and Ridge regressor decoder, with the latter achieving superior performance. We report neural reconstruction (zero-step-ahead) across folds and all the pixels within the brain areas for CEBRA. For continuous behavioral data, we report same-step decoding performance, and for categorical behavioral data, we report accuracy, auc, or F1-score as appropriate. The embedding dimension was explored from 1 to 256 in powers of 2. For widefield datasets, the "time-offset" was selected from $\{5, 10, 20\}$, while for the functional ultrasound imaging dataset, it was chosen from $\{3, 5, 10\}$. The "temperature" hyperparameter was varied within the set $\{0.01, 0.1, 1, 10\}$. The best-performing model across folds is reported for all experiments.

A.3.4. DPAD

DPAD (Dissociative Prioritized Analysis of Dynamics) (Sani et al., 2024) is a nonlinear dynamical model that focuses on learning behaviorally relevant neural dynamics and dissociating them from other dynamics in neural activity. It achieves this by fitting two dynamical models, formulated as a two-section RNN, one for behaviorally relevant neural dynamics and another for the remaining neural dynamics. DPAD also replaces linear mappings in the dynamical models with MLPs to flexibly learn the source of nonlinearity in the data. However, it is not specifically designed for image data and thus does not explicitly account for the spatial structure in the image-distributed neural data.

To compare with DPAD, we first identified the source of nonlinearity by using MLPs for each of the parameters of the model. We identified behavior readout parameter, $\boldsymbol{C}_z$, as source of nonlinearity and used an MLP with 1 or 2 hidden layers for this parameter. We tuned DPAD hyperparameters by varying the latent state dimension from 1 to 256 in powers of 2. For neural prediction and continuous behavioral data, we report one-step-ahead prediction for comparison. For categorical data, we report accuracy, AUC, or F1-score as appropriate.

A.3.5. STNDT

STNDT (Le & Shlizerman, 2022) utilizes a Transformer architecture for spatiotemporal modeling of neural population spiking activity. Originally designed for spiking data, we adapted STNDT to accept preprocessed LocaNMF features as input, as its direct application to raw images is computationally prohibitive due to the quadratic complexity of its spatial self-attention mechanism over a large number of pixels. For these LocaNMF features, we placed a Gaussian prior on the components and employed an MSE loss instead of the model's original Poisson likelihood. STNDT was trained using its original objectives, including masked reconstruction and a contrastive loss. For behavior decoding, we followed the approach discussed on STNDT's OpenReview forum, which is to use ridge regression to decode behavior from the learned latent states.

For hyperparameter tuning when using STNDT with LocaNMF features, we used the default hyperparameter choices such as number of transformers, masking ratio, etc., and varied the number of LocaNMF components provided as input (i.e., embedding dimension for STNDT), exploring values in the set $\{55, 123, 270\}$. Table A.5 reports performance of the adapted STNDT model which achieves the best behavior decoding.

A.3.6. TNDM

TNDM (Hurwitz et al., 2021) is a sequential autoencoder-based model designed to learn two distinct sets of latent factors from spiking data, with dimensionalities n_1 and n_2, corresponding to behaviorally relevant and behaviorally irrelevant dynamics, respectively. It achieves this by optimizing a combined neural-behavioral reconstruction loss. Given TNDM's original design for Poisson-distributed spiking data, we adapted it for our widefield imaging datasets. This involved using preprocessed LocaNMF features as input, assuming a Gaussian distribution for these input features, and changing TNDM's neural reconstruction loss to MSE.

Hyperparameter tuning for TNDM involved sweeping the dimensionalities for the behaviorally relevant latent factors, n_1, selected from $\{8, 16, 32, 64\}$, and the behaviorally irrelevant latent factors, n_2, selected from $\{0, 8, 16, 32, 64\}$. Table A.5 reports performance of the adapted TNDM model which achieves the best behavior decoding.

A.4. Datasets Details

A.4.1. Widefield Calcium Imaging (WFCI) Datasets

The WFCI datasets were collected from head-fixed mice performing a decision-making task, where they reported the spatial position of auditory or visual stimuli by licking the corresponding spout (Churchland et al., 2019). Neural activity across the dorsal cortex was optically recorded at 30 and 15 Hz for WFCI 1 and WFCI 2 datasets, respectively. We preprocessed the neural images to remove hemodynamic artifacts using a linear regression method (Musall et al., 2019; Valley et al., 2020). The raw neural images, with dimensions 540x640 pixels, were cropped to include only the brain regions and downsampled to 128x128 pixels. A pixel-wise temporal causal filter (0.1 Hz, 2nd order Butterworth high-pass) was applied to remove drift in the time series.

WFCI 1: This dataset consists of 248 trials with variable lengths (6.59 ± 0.50 seconds). Concurrently with neural recordings, behavior videos were recorded from two viewpoints (face and bottom; see Figure 2a). Following a similar procedure to (Musall et al., 2019), 14 dimensions of continuous behavior were extracted from seven regions of interest (eye, nose, whisker, paw, chest, body, and mouth) in the videos (see Figure 2c). For each region, the first principal component of both the original video frames and the motion video frames (computed as the absolute temporal derivative of frames) was extracted and used for behavioral prediction (see Figure A.5).

WFCI 2: This dataset comprises 412 trials with variable lengths (6.30 ± 0.37 seconds) with the same trial structure and neural recordings as WFCI 1. However, instead of behavior videos, four binary sensors detected contact with the animal's forepaws (handles) and tongue (spouts), providing categorical behavioral data for decoding (see Figure 2d), where 1's in any of the binary traces represents the time samples where the mouse was touching the corresponding sensor.

A.4.2. Functional Ultrasound Imaging (fUSI) Dataset

The fUSI dataset consists of recordings from a non-human primate performing a memory-guided saccade or reach task to either 2 or 8 peripheral targets (Griggs et al., 2023). Trials began with a 5 ± 1 second fixation period, followed by a 400 ms presentation of a peripheral cue. After the cue disappeared, there was a 5 ± 1 second memory period before the monkey executed a saccade or reach to the remembered target location. Successful trials were followed by a 1.5 ± 0.5 second hold period and then a reward. Each trial was followed by an inter-trial period before the next trial started.

Preprocessing: We applied a causal temporal voxel-wise filter (0.02 Hz high-pass Butterworth, 2nd order) to remove drift during the sessions. Similar to (Griggs et al., 2024), we z-scored the data voxel-wise over a rolling 60-frame buffer. Next, a pillbox spatial filter with a radius of 2 pixels was applied to each frame. The images were originally 128×132 pixels and cropped to 128×128 pixels.

Decoding: In this dataset, behavior consisted of the target the monkey reached or fixated on for each trial. Thus, we considered behavior as available only during the 1.5-second period (equivalent to 3 samples) before reward period when the monkey was fixating on the target. This gave us a categorical and intermittently recorded behavior time-series for modeling. We used these 3 samples as the only samples in the trials of length 30 where we have intermittent behavior available. To fit all variants of SBIND, we masked out other time points in the trial and optimized the parameters only for those 3 specific samples, effectively implementing intermittent behavior decoding during training. In the 2-directional tasks, we used binary target classification. In 8-directional tasks, similar to (Griggs et al., 2024), we used a multi-decoder approach in the decoder mapping to predict the vertical and horizontal directions. Thus, the decoder $\boldsymbol{D}^{(1)}$ has 6 softmaxed output dimensions: 3 for probabilities of left-right-center summing to 1, and 3 for probabilities of up-center-down summing to 1. For training PCA+LDA, we used either the 3 samples before the reward period, as in (Griggs et al., 2024), or all samples of the trial to decode directions. For training CEBRA, we used either the 3 samples before the reward period or all the samples (default choice for target classification task) of the trials for learning the embeddings, with the latter proving more effective for the target classification task. To fit DPAD, we used the 3 samples before reward period to fit the first RNN. During evaluation, we used the latent embedding at the last time-step in the trial to predict the direction of movement.

A.5. Supplementary Experiments

Table A.1. One-step-ahead behavior decoding and neural prediction performances for various ablations of SBIND across 5 folds for WFCI 1 dataset in terms of R^2. As indicated by the arrows, higher is better for R^2. For neural prediction, R^2 (Mean ± SEM) is reported across 5 folds and all pixels within the brain areas. For behavior decoding, R^2 (Mean ± SEM) is reported across 5 folds and 14 dimensions of behavior.

MODEL	PREPROCESSING	BEH. R^2 ↑	NEUR. R^2 ↑
MLP-SBIND	FLATTEN	0.3620 ± 0.0194	0.8209 ± 0.0033
MLP-SBIND	LOCANMF	0.4025 ± 0.0152	0.8702 ± 0.0015
MLP-SBIND	PCA	0.3934 ± 0.0145	$\mathbf{0.8926 \pm 0.0016}$
SBIND-UNSUP	-	0.4589 ± 0.0108	0.8724 ± 0.0039
SBIND NOATT	-	0.4612 ± 0.0107	0.8652 ± 0.0032
SBIND W/O $\boldsymbol{f_A}$	-	0.2080 ± 0.0453	0.8543 ± 0.0037
SBIND MSE $L_{\mathbf{Y}}$	-	$\mathbf{0.5030 \pm 0.0179}$	0.8217 ± 0.0133
SBIND	-	$\mathbf{0.5059 \pm 0.0166}$	$\mathbf{0.8724 \pm 0.0069}$

Table A.2. One-step-ahead behavior decoding and neural prediction performances for various ablations of SBIND across 5 folds for WFCI 2 dataset in terms of R^2 and AUC. As indicated by the arrows, higher is better for R^2 and AUC. For neural prediction, R^2 (Mean ± SEM) is reported across 5 folds and all pixels in the brain areas. For behavior decoding, AUC (Mean ± SEM) is reported across 5 folds and 4 classification tasks for left handle, right handle, left spout, and right spout.

MODEL	PREPROCESSING	BEH. AUC↑	NEUR. R^2↑
MLP-SBIND	FLATTEN	0.8706 ± 0.0038	0.7503 ± 0.0076
MLP-SBIND	LOCANMF	0.8823 ± 0.0037	0.6402 ± 0.0041
MLP-SBIND	PCA	0.8120 ± 0.0325	0.6703 ± 0.0239
SBIND-UNSUP	-	0.9182 ± 0.0039	$\mathbf{0.7970 \pm 0.0133}$
SBIND NOATT	-	0.9071 ± 0.0060	0.7451 ± 0.0043
SBIND W/O $\boldsymbol{f_A}$	-	0.8934 ± 0.0045	0.7339 ± 0.0022
SBIND MSE $L_{\mathbf{Y}}$	-	$\mathbf{0.9299 \pm 0.0029}$	0.7418 ± 0.0049
SBIND	-	$\mathbf{0.9282 \pm 0.0020}$	$\mathbf{0.7749 \pm 0.0074}$

Table A.3. Behavior decoding and neural prediction R^2 (Mean ± SEM) across folds for WFCI 1 dataset. As indicated by the arrows, higher is better for R^2. For neural prediction, R^2 (Mean ± SEM) is reported across 5 folds and all pixels in the brain areas. For behavior decoding, R^2 (Mean ± SEM) is reported across 5 folds and 14 dimensions of behavior.

MODEL	PREPROCESSING	BEH. R^2 ↑	NEUR. R^2 ↑
DPAD	FLATTEN	0.3826 ± 0.0189	0.8434 ± 0.0022
DPAD	LOCANMF	0.4128 ± 0.0133	0.8697 ± 0.0011
DPAD	PCA	0.3839 ± 0.0157	$\mathbf{0.8902 \pm 0.0008}$
CEBRA	FLATTEN	0.4001 ± 0.0132	0.5957 ± 0.0216
CEBRA	LOCANMF	0.3745 ± 0.0081	0.4453 ± 0.0099
CEBRA	PCA	0.3686 ± 0.0127	0.4638 ± 0.0079
SBIND	-	$\mathbf{0.5059 \pm 0.0166}$	$\mathbf{0.8724 \pm 0.0069}$

Table A.4. Behavior decoding AUC and neural prediction R^2 (Mean $\pm$ SEM) across folds for WFCI 2 dataset. As indicated by the arrows, higher is better for R^2 and AUC. For neural prediction, R^2 (Mean $\pm$ SEM) is reported across 5 folds and all pixels in the brain areas. For behavior decoding, AUC (Mean $\pm$ SEM) is reported across 5 folds and 4 classification tasks for left handle, right handle, left spout, and right spout. For CEBRA, a "KNNDecoder" is used for decoding which does not directly report AUC.

MODEL	PREPROCESSING	BEH. AUC↑	NEUR. R^2↑
DPAD	FLATTEN	0.8782 ± 0.0059	0.7440 ± 0.0049
DPAD	LOCANMF	0.8888 ± 0.0057	0.6374 ± 0.0033
DPAD	PCA	0.8039 ± 0.0090	0.7182 ± 0.0038
CEBRA	FLATTEN	-	0.7228 ± 0.0052
CEBRA	LOCANMF	-	0.4971 ± 0.0045
CEBRA	PCA	-	0.4913 ± 0.0131
SBIND	-	$\mathbf{0.9282 \pm 0.0020}$	$\mathbf{0.7749 \pm 0.0074}$

Table A.5. Comparison of baselines including adapted STNDT and TNDM on WFCI1 dataset. Behavior decoding and neural prediction MSE and R^2 (Mean ± SEM) across folds.

MODEL	PREPROCESSING	NEUR. MSE ↓	NEUR. R^2 ↑	BEH. MSE ↓	BEH. R^2 ↑
DPAD	LOCANMF	0.0543 ± 0.0009	0.8697 ± 0.0011	0.5877 ± 0.0226	0.4128 ± 0.0133
CEBRA	LOCANMF	0.4976 ± 0.0241	0.4453 ± 0.0099	0.6250 ± 0.0194	0.3745 ± 0.0081
STNDT	LOCANMF	0.0685 ± 0.0090	0.8376 ± 0.0088	0.6033 ± 0.0240	0.3951 ± 0.0156
TNDM	LOCANMF	0.7912 ± 0.0290	0.5022 ± 0.0081	0.7749 ± 0.0240	0.2233 ± 0.0109
SBIND-UNSUP	-	$\mathbf{0.0403 \pm 0.0020}$	$\mathbf{0.8724 \pm 0.0039}$	0.5413 ± 0.0185	0.4589 ± 0.0108
SBIND	-	$\mathbf{0.0414 \pm 0.0029}$	$\mathbf{0.8724 \pm 0.0069}$	$\mathbf{0.4955 \pm 0.0254}$	$\mathbf{0.5059 \pm 0.0166}$

Table A.6. Performance comparison of SBIND on WFCI1 datasets using two recurrent update formulations for integrating current neural image information ($\mathbf{Y}_k$). The table contrasts the concatenation approach as in Eq. A.12, where the encoded input $\boldsymbol{K}(\mathbf{Y}_k)$ is concatenated with the latent state $\mathbf{X}_k$ before the recurrent function, against the summation approach as in Eq. A.1. Results demonstrate that the concatenation method Eq. A.12 yields improved neural prediction MSE, neural R^2, behavioral MSE, and behavioral R^2. for WFCI1 dataset.

Model Formulation	Neural MSE	Neural R^2	Beh MSE	Beh R^2
SBIND w. Recurrent Eq. A.12	0.0414 ± 0.0029	0.8724 ± 0.0069	0.4955 ± 0.0254	0.5059 ± 0.0166
SBIND w. Recurrent Eq. A.1	0.0664 ± 0.0013	0.8545 ± 0.0027	0.5306 ± 0.0198	0.4680 ± 0.0182

Table A.7. Comparison of various ablations in behavior decoding accuracy (quantified as proportion of trials whose target was correctly decoded) and AUC across 10 folds and all sessions of fUSI Data. For 8-directional sessions a multi-decoder approach is used with two decoders to predict vertical and horizontal directions (left-right-stationary). Multi-class AUC averaged over two vertical and horizontal directions, 4 sessions and 10 folds are reported. As indicated by the arrows, higher is better for accuracy and AUC.

		2-DIRECTIONAL SESSIONS		8-DIRECTIONAL SESSIONS	
MODEL	PREPROCESSING	BEH. ACCURACY↑	BEH. AUC↑	BEH. ACCURACY↑	BEH. AUC↑
MLP-SBIND	FLATTEN	0.5984 ± 0.0162	0.6285 ± 0.0216	0.2175 ± 0.0169	0.5833 ± 0.0196
MLP-SBIND	PCA	0.6565 ± 0.0178	0.7296 ± 0.0214	0.2966 ± 0.0183	0.6823 ± 0.0170
SBIND-UNSUP	-	0.7030 ± 0.0180	0.7859 ± 0.0194	0.3411 ± 0.0187	0.7304 ± 0.0176
SBIND NOATT	-	0.6889 ± 0.0184	0.7480 ± 0.0237	0.2893 ± 0.0179	0.6975 ± 0.0145
SBIND	-	$\mathbf{0.7300 \pm 0.0191}$	$\mathbf{0.8067 \pm 0.0180}$	$\mathbf{0.3521 \pm 0.0201}$	$\mathbf{0.7393 \pm 0.0169}$

Table A.8. Comparison of various ablations of SBIND in one-step-ahead neural prediction performance (MSE and R^2) across 10 folds and all sessions of fUSI dataset. As indicated by the arrows, lower is better for MSE and higher is better for R^2.

		2-directional sessions		8-directional sessions	
Model	Preprocessing	Neur. MSE↓	Neur. R^2↑	Neur. MSE↓	Neur. R^2↑
MLP-SBIND	Flatten	0.7885 ± 0.0067	0.2641 ± 0.0060	0.8743 ± 0.0065	0.2274 ± 0.0071
MLP-SBIND	PCA	0.6322 ± 0.0054	0.3985 ± 0.0043	0.7058 ± 0.0030	0.3586 ± 0.0038
SBIND-Unsup	-	$\mathbf{0.4453 \pm 0.0127}$	$\mathbf{0.6029 \pm 0.0112}$	$\mathbf{0.3827 \pm 0.0086}$	$\mathbf{0.6683 \pm 0.0073}$
SBIND NoAtt	-	0.5036 ± 0.0155	0.5545 ± 0.0133	0.4230 ± 0.0109	0.6344 ± 0.0090
SBIND MSE $L_{\mathbf{Y}}$	-	0.4900 ± 0.0108	0.5555 ± 0.0094	0.4225 ± 0.0076	0.6287 ± 0.0064
SBIND	-	$\mathbf{0.4725 \pm 0.0165}$	$\mathbf{0.5736 \pm 0.0144}$	$\mathbf{0.3919 \pm 0.0107}$	$\mathbf{0.6558 \pm 0.0094}$

Table A.9. Comparison of baselines in behavior decoding AUC and neural prediction R^2 across 10 folds and all sessions (Mean ± SEM). For DPAD and SBIND, in 2-directional sessions the AUC for binary classification is reported over 10 folds and 9 sessions of fUSI Data. In 8-directional sessions, a multi-decoder approach is used with two decoders to predict vertical and horizontal directions (left-right-stationary). Multi-class AUC averaged over two vertical and horizontal directions, 4 sessions, and 10 folds are reported. For CEBRA, a "KNNDecoder" is used for decoding which does not directly report AUC. As indicated by the arrows, higher is better for AUC and R^2.

		2-directional sessions		8-directional sessions	
Model	Preprocessing	Beh. AUC↑	Neur. R^2↑	Beh. AUC↑	Neur. R^2↑
LDA	PCA	0.7130 ± 0.0242	-	0.6987 ± 0.0164	-
DPAD	Flatten	0.5940 ± 0.0221	0.2355 ± 0.0066	0.6060 ± 0.0157	0.2011 ± 0.0079
DPAD	PCA	0.7399 ± 0.0208	0.3938 ± 0.0045	0.6752 ± 0.0179	0.3554 ± 0.0040
CEBRA	Flatten	-	-0.3839 ± 0.0128	-	-0.3518 ± 0.0085
CEBRA	PCA	-	-0.2731 ± 0.0069	-	-0.3076 ± 0.0103
SBIND	-	$\mathbf{0.8067 \pm 0.0180}$	$\mathbf{0.5736 \pm 0.0144}$	$\mathbf{0.7393 \pm 0.0169}$	$\mathbf{0.6558 \pm 0.0094}$

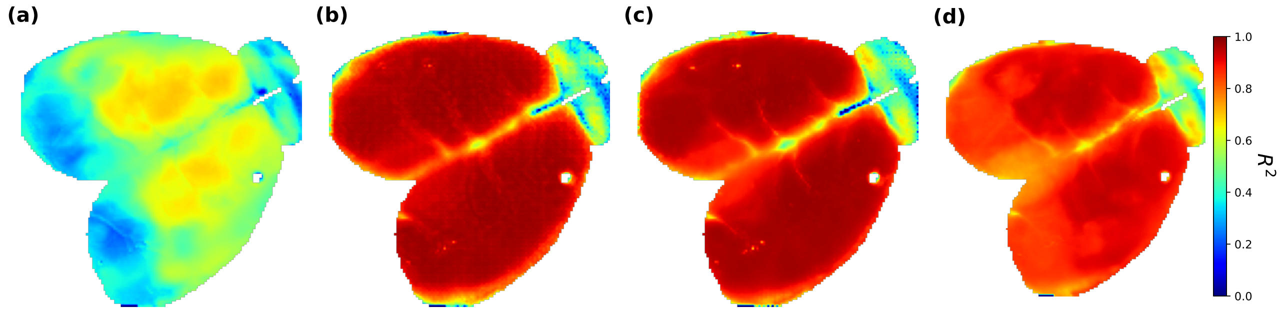

Figure A.2. **Pixel-wise neural prediction R^2 values across brain regions.** Neural prediction maps for the WFCI 1 dataset are depicted for **(a)** CEBRA, **(b)** SBIND, **(c)** SBIND NoAtt, and **(d)** DPAD. SBIND produces more detailed neural predictions compared to its variant without the self-attention mechanism. CEBRA poorly predicts neural activity because it uses latent embeddings guided by behavior and lacks extra embedding dimensions for residual neural activity unrelated to behavior. This is consistent with the observation that widefield calcium imaging datasets often contain significant neural activity unrelated to behavior (Musall et al., 2019). Higher is better for R^2.

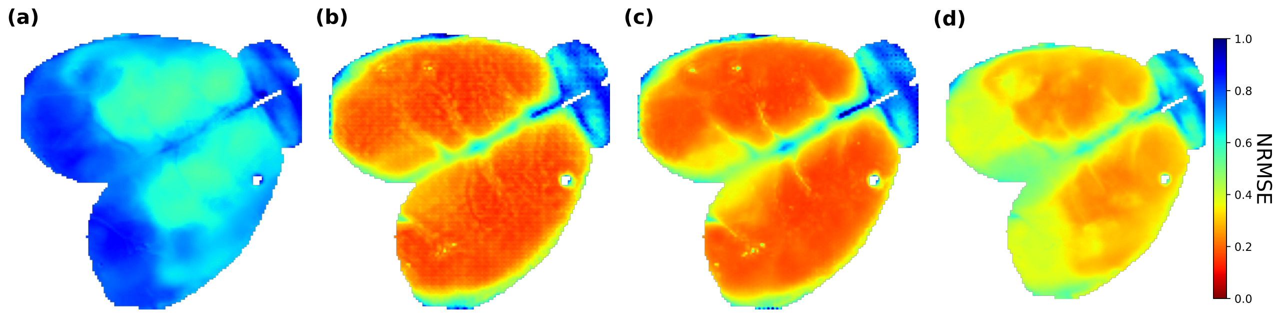

Figure A.3. **Pixel-wise neural prediction NRMSE values across brain regions.** Neural prediction maps for the WFCI 1 dataset are depicted for **(a)** CEBRA, **(b)** SBIND, **(c)** SBIND NoAtt, and **(d)** DPAD. Lower is better for NRMSE.

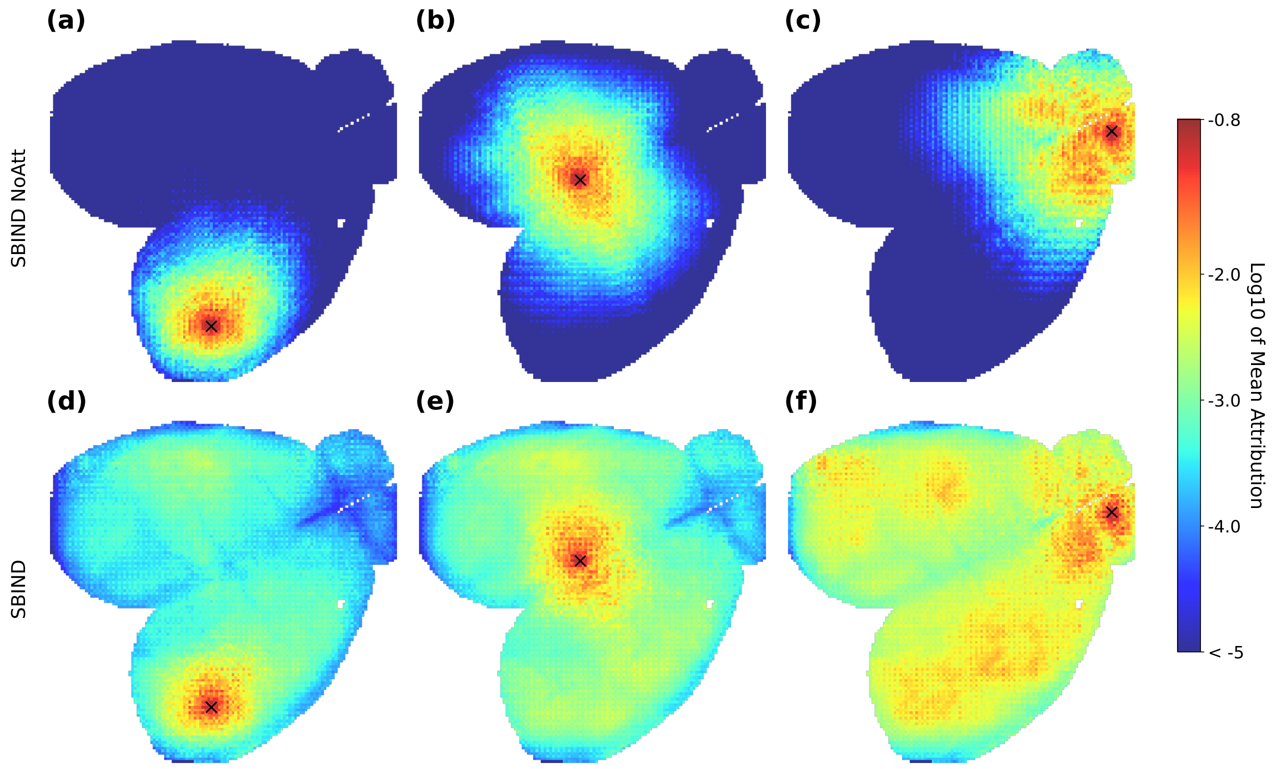

Figure A.4. **Mean contribution of all brain regions to predicting the activity of three different pixels marked by × in the brain map.** The plots display the mean attribution of whole-brain activity to the neural prediction of specific points, derived using the Captum framework. We calculated the attribution of each input image across all time points to the neural prediction of the specified points in different plots. These attribution maps were then averaged across time for different neural images to find the mean attribution. This analysis was performed on the test data from the WFCI 1 dataset after training both models. **(a-c)** SBIND NoAtt. **(d-f)** SBIND.

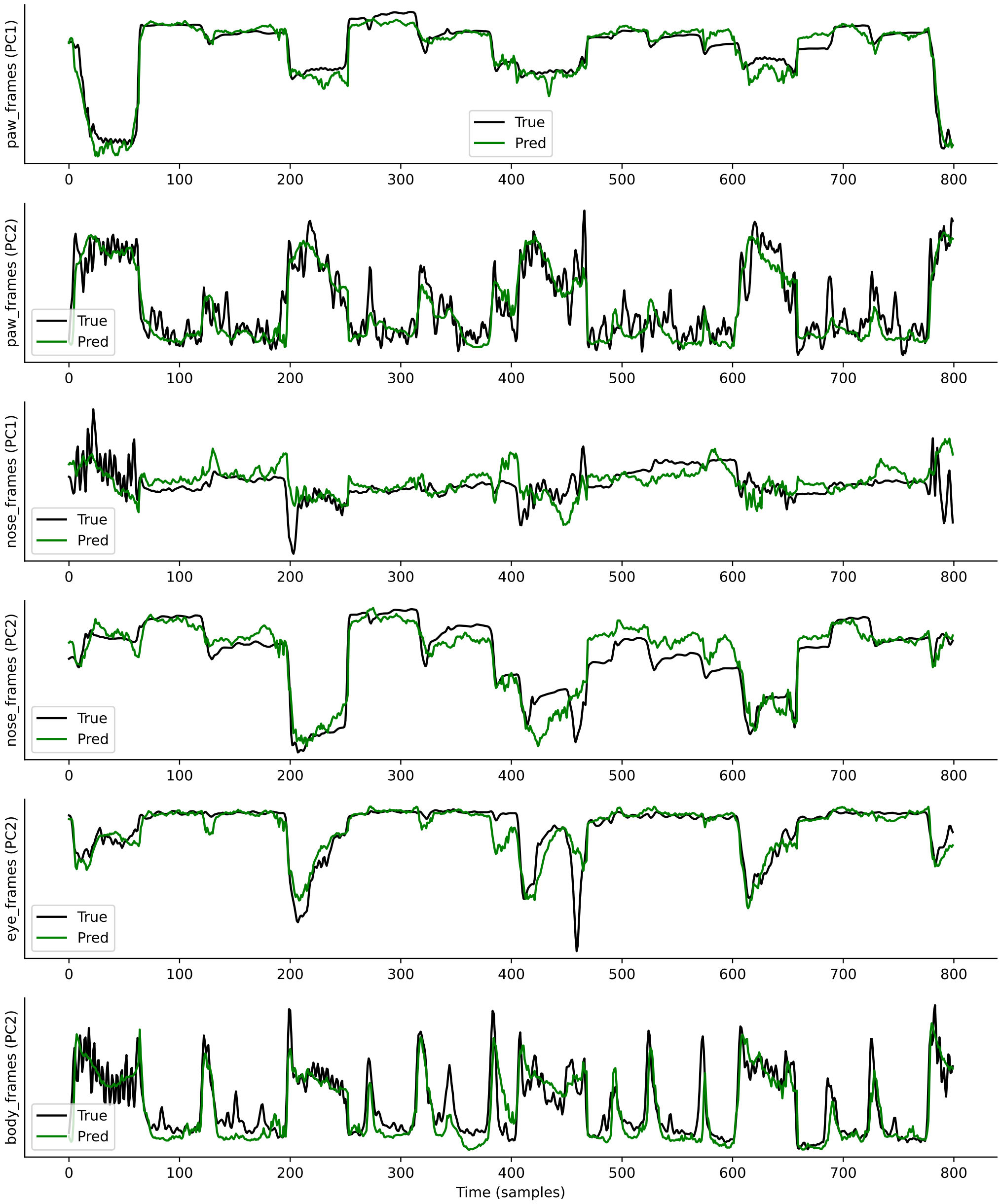

Figure A.5. **SBIND example behavior decoding.** Predictions over 6 dimensions of continuous behavior extracted from behavior videos for WFCI 1 dataset. (See Appendix A.4 and Figure 2 for details of behavior extraction.)

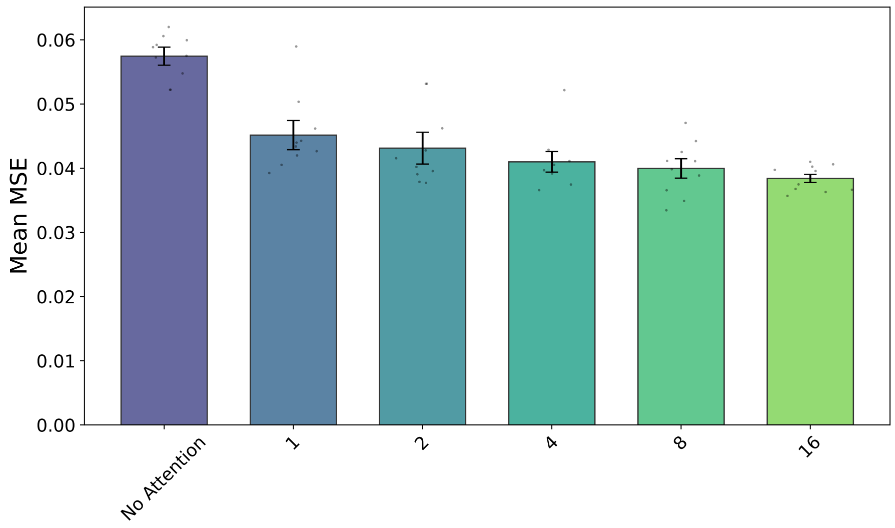

Figure A.6. **Larger Self-Attention Patch Sizes Lead to Better Neural Prediction.** Neural prediction MSE across different patch sizes for the WFCI 1 dataset across 5 folds and 2 runs.
